# Surface Roughness Effects on Self-Interacting and Mutually Interacting Rayleigh Waves

**DOI:** 10.3390/s21165495

**Published:** 2021-08-15

**Authors:** Chaitanya Bakre, Cliff J. Lissenden

**Affiliations:** Department of Engineering Science and Mechanics, The Pennsylvania State University, University Park, PA 16802, USA; cxb666@psu.edu

**Keywords:** Rayleigh waves, surface roughness, system nonlinearity

## Abstract

Rayleigh waves are very useful for ultrasonic nondestructive evaluation of structural and mechanical components. Nonlinear Rayleigh waves have unique sensitivity to the early stages of material degradation because material nonlinearity causes distortion of the waveforms. The self-interaction of a sinusoidal waveform causes second harmonic generation, while the mutual interaction of waves creates disturbances at the sum and difference frequencies that can potentially be detected with minimal interaction with the nonlinearities in the sensing system. While the effect of surface roughness on attenuation and dispersion is well documented, its effects on the nonlinear aspects of Rayleigh wave propagation have not been investigated. Therefore, Rayleigh waves are sent along aluminum surfaces having small, but different, surface roughness values. The relative nonlinearity parameter increased significantly with surface roughness (average asperity heights 0.027–3.992 μm and Rayleigh wavelengths 0.29–1.9 mm). The relative nonlinearity parameter should be decreased by the presence of attenuation, but here it actually increased with roughness (which increases the attenuation). Thus, an attenuation-based correction was unsuccessful. Since the distortion from material nonlinearity and surface roughness occur over the same surface, it is necessary to make material nonlinearity measurements over surfaces having the same roughness or in the future develop a quantitative understanding of the roughness effect on wave distortion.

## 1. Introduction

Many types of structures suffer damage due to rigorous operating and environmental conditions. Various degradation mechanisms such as fatigue, corrosion, and strength reduction can cause the failure of components, which may degrade structural performance or lead to catastrophic failure and life-threatening situations. Inspecting the structural integrity of mechanical components using nondestructive evaluation (NDE) techniques or structural health monitoring (SHM) techniques is crucial. Rayleigh waves, and surface acoustic waves (SAW) in general, are highly effective for surface inspections as their energy is concentrated near the surface [1]. The linear parameters of Rayleigh waves, such as the wave speed and the attenuation, have been effectively used to detect evolution of the material properties [2,3,4,5]. Rayleigh wave speed has a strong dependence on porosity [6], while attenuation depends on various factors, including absorption, diffraction, and scattering caused by voids, pores, inclusions, and grain boundaries [7,8].

Likewise, the nonlinearity of Rayleigh waves has been leveraged for detecting changes in the material or material microstructure that lead to macroscale damage [9]. The interaction of Rayleigh waves with the microstructure results in distortion of the waves and generation of higher harmonics. The relative nonlinearity parameter (to be defined subsequently) for Rayleigh waves depends on the spectral amplitudes at the primary and second harmonic frequencies. The relative nonlinearity parameter of Rayleigh waves is the following:

- effective in detecting fatigue cracking at an early stage [10,11];
- sensitive to plastic deformation, cold work, and residual stress [12];
- able to distinguish different aluminum alloys in pristine states based on their material nonlinearity due to lattice anharmonicity [13];
- sensitive to precipitate hardening due to heat treatments [14], thermal embrittlement [15,16], sensitization of stainless steel [17], and stress corrosion cracking [18].

Both linear and nonlinear Rayleigh wave measurements require sensors that send and receive the waves at ultrasonic frequencies. Recent studies of Rayleigh wave measurements include Ghafoor et al. [19], Li et al. [20], Song et al. [21], Li et al. [22], and Sarris et al. [23]. Many types of sensors can be used for this purpose including angle-beam, comb, interdigitated, and pulsed lasers. Understanding the sensor data, especially when using the relative nonlinearity parameter, is an important first step for NDE and SHM.

In the above-mentioned applications of Rayleigh waves, the researchers are careful to make measurements on smooth surfaces because roughness is known to affect the propagation characteristics of Rayleigh waves. Surface roughness in the Rayleigh wave transmission path causes scattering, which induces attenuation and dispersion [24,25,26,27,28]. Urazakov and Fal’kovskii [28] and Maradudin and Mills [25] first analytically studied the attenuation effects of surface roughness on Rayleigh wave propagation using Rayleigh’s method and a Green’s function method. The authors limit the amplitude of roughness to be sufficiently small compared to the Rayleigh wavelength in order to use perturbation theory. The surface irregularities act as scatterers causing mode conversion to bulk waves or other Rayleigh waves. Both approaches predict the Rayleigh wave attenuation to be primarily caused by mode conversion to bulk waves as opposed to Rayleigh waves in other directions. The studies also indicate that the attenuation is proportional to the fifth power of the frequency. Steg and Klemens [29] arrived at the same relationship between attenuation and frequency using the method of mass defects. De Billy et al.’s [30] attenuation measurements on duraluminum samples revealed the same fifth power dependence of attenuation on frequency, validating the theoretical predictions in [25] and [26].

De Billy et al. [30] also noticed a reduction in Rayleigh wave speed for one-dimensional surface roughness. Later, using Rayleigh’s method, Eguiluz and Maradudin [27] obtained the dispersion relation for Rayleigh waves due to surface irregularities. Sinclair [31] used the method of mass loading on a smooth surface to obtain the frequency dependence of Rayleigh wave speed along rough surfaces. Krylov and Smirnova [24] also experimentally studied the dispersion effects of Rayleigh waves on rough surfaces and found that the surface roughness caused a reduction in the Rayleigh wave speed, and the decrease in speed increased with increasing frequency. The authors reported that the frequency dependence of the attenuation agrees with the theoretical models discussed by Eguiluz and Maradudin [27] and Huang and Maradudin [26]. A variation of 0.5–1.5% in the frequency-dependent velocity was observed for surface roughness with an RMS (root mean square) surface height deviation of 17 μm in the frequency range 1 to 4 MHz.

More recently, the adverse effect caused by surface roughness was studied relative to Rayleigh wave based residual stress measurement for a shot peening operation [32,33]. The dispersion caused by the surface roughness rendered a large deviation in the measurement of residual stress. In related research, Liu et al. [12] observed a decrease in the relative nonlinearity parameter from 81% to 44.5% when the rough shot-peened specimen was hand polished using emery paper (grit # 600, 800, 1200). However, very limited literature is available that accounts for the effect of surface roughness on the nonlinear characteristics of Rayleigh waves.

Detection of Rayleigh wave distortion associated with material nonlinearity can be a powerful tool for NDE and SHM, but since the wave distortion is typically small, it is necessary to well understand the other nonlinearities that creep into the measurement. The effect of attenuation on nonlinear Rayleigh waves has been accounted for by Cantrell [34], but it has not been applied to the surface roughness problem.

This paper reports on Rayleigh wave propagation in a thick 7075 aluminum block. The objective of the paper is to assess the effect that surface roughness has on the distortion of Rayleigh waves. Three specimens of the same material with different surface roughness are used to investigate the effects of surface roughness on the relative nonlinearity parameter for the second harmonic and mutually interacting Rayleigh waves. The single-frequency and dual-frequency Rayleigh waves are generated using angle beam transducers and received using a laser receptor. In this paper, the nonlinearity at various points in the sensing system are measured, viz. output from the amplifier, output from the transducer, and output from the wedge used for the angle beam transducer. Second, two different methods for the generation of dual-frequency Rayleigh waves are examined for their effectiveness in studying the mutual interaction, viz. using a single transducer attached to the wedge and using two adjacently placed wedge-transducers. Then, the attenuation coefficients are obtained for the three specimens with different surface roughness values. Finally, the measured and attenuation-corrected relative nonlinearity parameters are compared to understand the roughness effects on the Rayleigh wave distortion.

## 2. Materials and Methods

**Figure 1:**
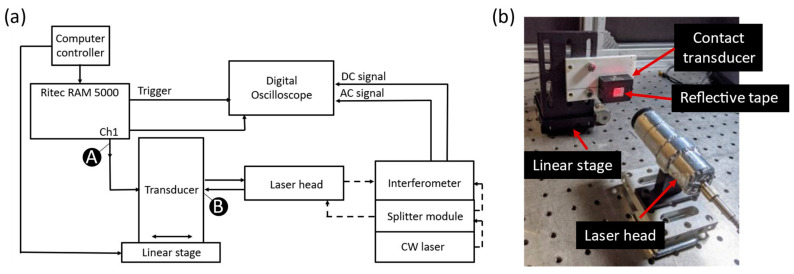
Test setup for measurement of system nonlinearity: (**a**) Block diagram where solid and dashed lines represent electrical cables and optical fibers respectively, (**b**) Photograph of the laser head illuminating reflective tape on the transducer surface.

The experimental setup used to investigate the effect of surface roughness on nonlinear Rayleigh waves consists of an angle-beam transducer for the generation of Rayleigh waves on an aluminum alloy specimen and an adaptive interferometer for their reception. Toneburst excitations at single and dual frequencies enable the investigation of nonlinearity from self-interaction as well as from mutual interaction. We start characterizing the nonlinearity of the sensing system by receiving the vibratory response of the transducer itself by impinging the reception laser beam directly on the transducer surface, as shown in the block diagram and photograph in Figure 1.

Contact transducers (Benchmark series 113-244-591, 113-863-600, or 113-232-591; Baker Hughes, Houston, TX, USA) are actuated by a gated amplifier (RAM-5000 SNAP, Ritec Inc., Warwick, RI, USA). These transducers have center frequencies of 2.25, 3.5, and 5.0 MHz, respectively. The transducer is mounted on a linear stage to enable focusing the laser interferometer on the surface of the transducer. Retroreflective tape is applied on the surface of the transducer to improve the reflectivity. An adaptive laser interferometer measures the out-of-plane displacement from the surface of the transducer. The received signals are observed using an oscilloscope and recorded for post-processing.

The laser interferometer (AIR-1550-TWM, Intelligent Optical Systems Inc., Torrance, CA, USA), used to measure the out-of-plane surface displacements, is comprised of four components: (1) a 1550 nm continuous wave (CW) laser with the maximum power capacity of 2 W, (2) a splitter module, (3) a laser head, (4) and an interferometer. The laser beam is delivered by an optical fiber. The splitter module divides the CW laser beam into a reference beam and a probe beam. An optical fiber delivers the probe beam to the laser head, which uses a collimating lens pair to focus it on the surface of the sample. The out-of-plane surface displacements distort the probe beam. The distorted probe beam reflected from the surface is re-captured by the laser head. The distorted probe beam and the reference beam are combined in a photorefractive material inside the interferometer. The photorefractive material generates a time-varying voltage that is proportional to the instantaneous surface displacements. The photorefractive material also inherently rejects slowly-varying changes (<10 kHz) typical of low-frequency background noise.

The laser interferometer provides two outputs, viz. an AC signal and a DC level, that are recorded on an oscilloscope (InfiniiVision MSOX3024T, Keysight, Santa Rosa, CA, USA). The AC signal contains the time-varying voltage proportional to the surface displacements, while the DC level provides a measure of the received light reflected from the surface. The amount of light received by the laser head depends mainly on the power of the incident probe beam, the reflectivity and roughness of the surface, and the position of the laser head relative to the surface. Thus, normalizing the AC signal by the DC level provides a means to compare the signals obtained from rough surfaces (that scatter the laser beam) with those obtained from smooth surfaces. In this research, the received AC signals are normalized by the corresponding DC level.

**Figure 2:**
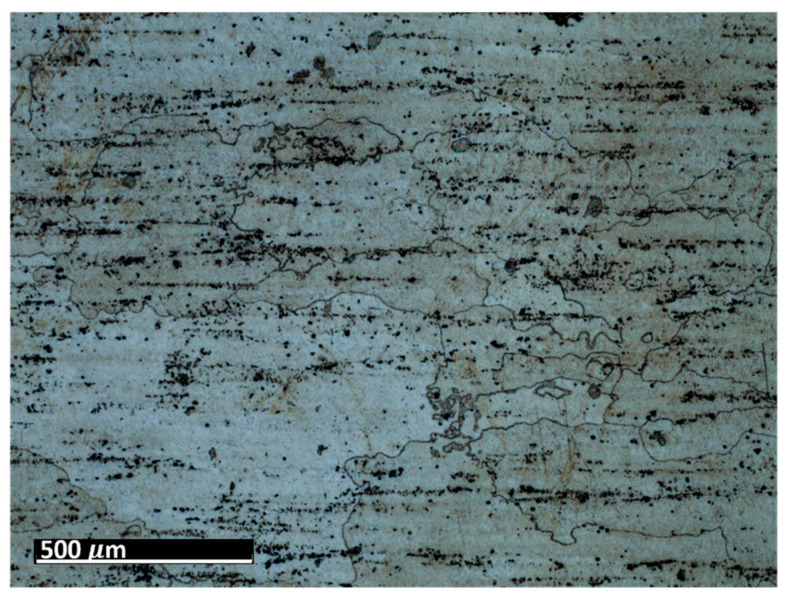
Optical microscope (Zeiss SmartZoom) image of polished and etched (Kroll’s reagent) aluminum block surface. Pancake-type grains and a distribution of fine precipitates are apparent.

The test specimens are 7075 aluminum blocks 170 mm × 40 mm ×20 mm having different surface roughness values. Each block is made from the same material, for which the microstructure is shown in Figure 2. The length and width of the elongated grains in μm are (509 ± 16, 266 ± 10), (559 ± 16, 225 ± 9), (547 ± 15, 207 ± 10) for samples 1, 2, and 3, respectively. The hardness values are 111HV0.5, 112HV0.5, and 114HV5 for samples 1, 2, and 3, respectively. The moderate and rough surface samples are obtained by performing a three-pass and a single pass wire-cut EDM (M500S, Seibu Electric and Machinary, Koga, Japan) operation. The smooth surface is obtained by whetstone polishing. The surface roughness is characterized using a white light interferometer (NexView 9000, Zygo, Middlefield, CT, USA) and quantified using Gwyddion, which is an open source software for Statistical Parametric Mapping (SPM) data analysis [35]. A 50× Mirau objective is used to achieve an optical resolution of 0.52 μm in the *x* and *y* directions based on the Sparrow criteria (Optical resolution = 0.5 λ/NA, where λ = 570 nm and NA = 0.55). The spatial sampling based on the camera pixel size is 0.17 μm and the area of the inspected region is 170 μm × 170 μm. Table 1 gives the 3D and 1D surface profiles for the three test blocks. While Deltombe et al. [36] describe a procedure to determine which surface roughness parameters are most relevant for a specific application, we simply provide the linear parameters (ISO 4287): *P_a_* (arithmetic average), *P_q_* (root mean square), and *P_t_* (peak-to-valley distance), and areal parameters (ISO 25178-2): $S_{a}\;$(arithmetic mean height), $S_{q}$ (root mean square height), $S_{z}$ (maximum height), and $S_{dq}$ (root mean square gradient). The linear and areal surface roughness parameters for each sample are tabulated in Table 2. The mean values are calculated from 1022 measurements. The surface roughness can affect the generation, wave propagation, as well as the reception of Rayleigh waves. However, this paper focuses on the effect of surface roughness on nonlinear Rayleigh wave propagation. This is much different than bulk waves reflecting from a rough surface as in Wang et al. [37]. Therefore, the specimen surface where the wedge is coupled is made smooth by sequential abrasion with emery paper (grit #400, 600, 800, 1000, 1500). This ensures that there is no influence of the surface roughness on the Rayleigh wave generation. In contrast, the surface where the Rayleigh waves are received is not polished. But as mentioned before, the laser interferometer used in this study is adaptive to the varying surface roughness and enables factoring out the effects of surface roughness on reception.

The output level of the gated amplifier is varied from 20–80% in increments of 10% to increase the wave amplitude to determine the nonlinearity parameter. Finally, the Plexiglas wedge is coupled to the block with ultrasonic gel (Soundsafe, Sonotech, State College, PA, USA) and preloaded by a spring force.

### 2.1. Relative Nonlinearity Parameter

In this study the relative nonlinearity parameter is used as a relative measure to compare the effect of surface roughness on the self-interaction and mutual interaction of Rayleigh waves. The relative nonlinearity parameter for second harmonic generation (from self-interaction) is typically defined to be

(1) $$\beta^{\prime }=\frac{A_{2}}{A_{1}^{2}}$$

where $A_{1}$ and $A_{2}$ are the spectral amplitudes at the primary and second harmonic frequencies respectively. The generalized definition of the relative nonlinearity parameter for mutual interaction of waves at the primary frequencies $f_{a}\;\leq \;f_{b}$ used herein is

(2) $$\beta^{\prime }=\frac{A_{\left(f_{b}\pm f_{a}\right)}}{A_{f_{a}}A_{f_{b}}}$$

where the plus sign is associated with the sum frequency and the minus sign is associated with the difference frequency. If $f_{a}=f_{b}$ we have self-interaction instead of mutual interaction and Equation (2) gives the second harmonic in the case of the sum, and the quasi-static pulse at zero frequency in the case of the difference. To compute the relative nonlinearity parameter $\beta^{\prime }$, $A_{\left(f_{b}\pm f_{a}\right)}$ is plotted as a function of $A_{f_{a}}A_{f_{b}}$ as the output level of the amplifier is increased. For the range of output levels where the graph is linear, $\beta^{\prime }$ is obtained by linear regression.

### 2.2. Self-Interaction of Rayleigh Waves

**Figure 3:**
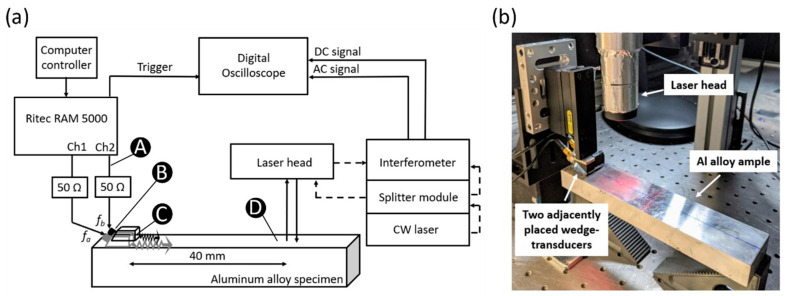
Rayleigh wave test setup: (**a**) Block diagram where solid and dashed lines represent electrical cables and optical fibers respectively, (**b**) Adjacent angle-beam transducers actuate dual-frequency Rayleigh waves, which are received by the laser head.

When conducting nonlinear ultrasonic testing to assess the material nonlinearity, it is important to know what other nonlinearities are embedded in the measurements. In this work the nonlinearity of the sensing system is investigated by analyzing the signal in the sensing system at the points shown in Figure 3:

- Point A—amplifier output monitoring point
- Point B—surface of the transducer, measured by laser interferometer
- Point C—surface of the wedge, measured by laser interferometer
- Point D—surface of the specimen, measured by laser interferometer.

The primary frequency used for system nonlinearity assessment is *f*_0_ = 5 MHz, therefore the second harmonic occurs at 10 MHz.

The surface roughness effects on the self-interaction of Rayleigh waves are studied for the primary frequencies 2, 3.5, and 5 MHz, and the relative nonlinearity parameter are obtained on the three aluminum blocks with different surface roughness. The attenuation coefficients are obtained for the excitation frequencies and the respective second harmonic frequencies to check the veracity of the attenuation correction that accounts for the surface roughness effects on the relative nonlinearity parameter. The laser head is thus scanned from 30 mm to 130 mm from the angle beam transducer along the wave propagation direction, and the measurements are obtained in 5 mm increments.

### 2.3. Mutual Interaction of Rayleigh Waves

The mutual interaction of waves at primary frequencies $f_{a}$ = 3.2 MHz and $f_{b}$ = 3.84 MHz generated by a single transducer is studied. Note that the two frequencies are selected close to the nominal central frequency of the transducer. The peak amplitudes of the two tonebursts are equal, and their relative phase difference is zero. The second-order frequencies are: $f_{b}-f_{a}=0.64$ MHz, $2f_{a}=6.4$ MHz, $f_{b}+f_{a}=7.04$ MHz, and $2f_{b}=7.68$ MHz. When operated in the ‘combine modulation’ mode, the gated amplifier provides a dual-frequency toneburst signal on Channel 1. The signals are obtained at Point A and Point B, as shown in Figure 1.

**Figure 4:**
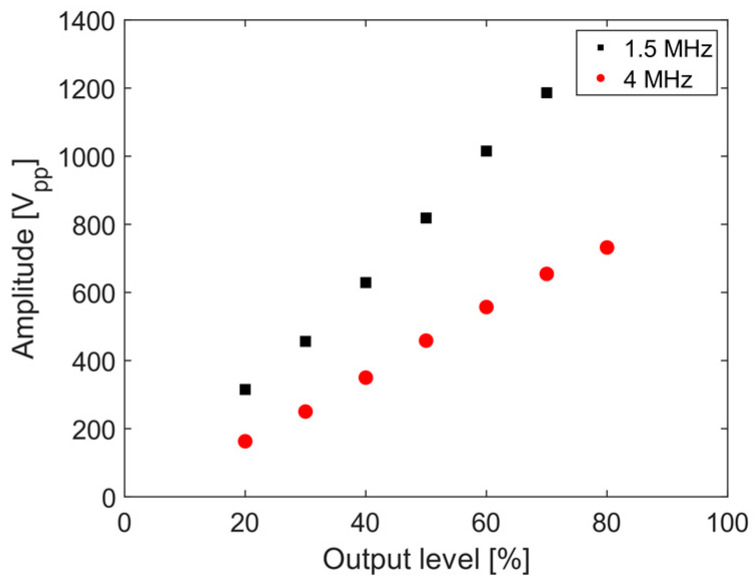
Amplitude of toneburst signal sent to the transducer as a function of amplifier output level for center frequencies of 1.5 and 4.0 MHz.

For the adjacently placed wedge-transducers, the wave mixing occurs due to ultrasonic beam spreading. The use of two transducers allows for a wider selection of excitation frequencies. The signal being sent to the piezoelectric transducer is monitored, and Figure 4 shows the peak-to-peak voltages as a function of output level supplied to the transducers for 1.5 and 4.0 MHz toneburst signals. This method avoids the intermodulation distortion effect as each transducer is excited by a toneburst signal having a single central frequency. Although the system nonlinearity contributes higher harmonics, the mutual interaction between the waves, which at second order occurs at the sum and difference frequencies, is not convoluted by system nonlinearities.

### 2.4. Signal Processing

1024 signals were synchronously averaged together and then recorded using the oscilloscope. The signals are normalized with respect to the DC level. Matlab algorithms are developed for further processing the recorded signals. A Hanning window is applied to the signal before computing the spectrum. The sampling frequency of the time record is 1.45 GHz. Zero-padding is used to improve the frequency resolution before the Fast Fourier Transform (FFT) function in Matlab is applied. The output of the Matlab FFT function is scaled by the time increment ($dt=6.9\times 10^{-10}\;s$) to obtain the linear spectrum.

## 3. Results

### 3.1. Sensing System Nonlinearity

**Figure 5:**
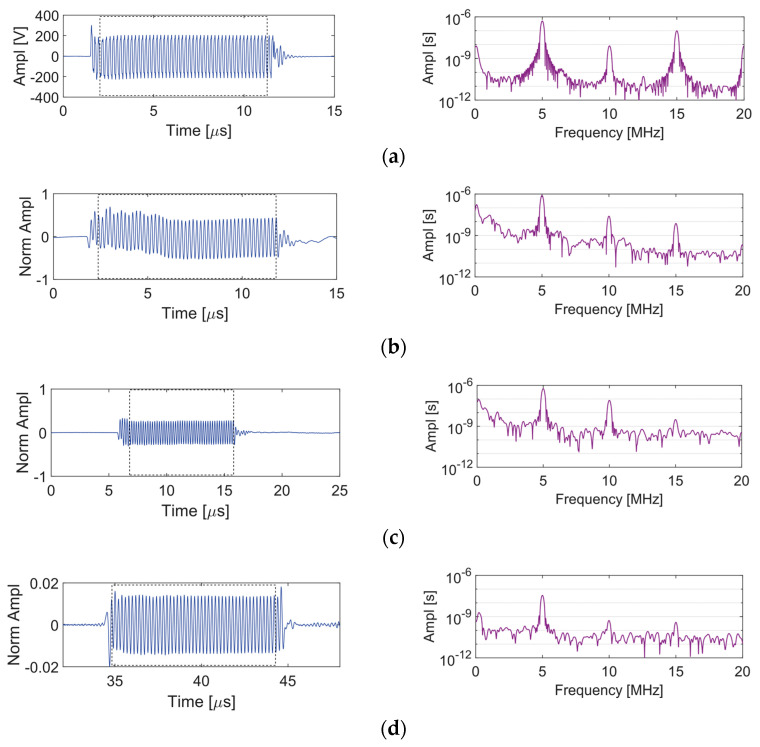
A-scans and frequency spectra for 5 MHz toneburst excitation at the 75% output level: (**a**) Point A, (**b**) Point B, (**c**) Point C, and (**d**) Point D.

**Figure 6:**
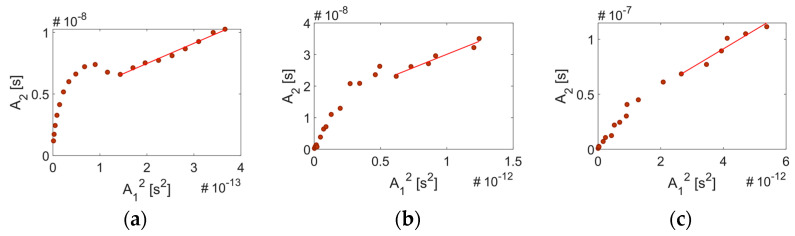
Linear regression to determine the relative nonlinearity parameter for the sensing system given a 5 MHz signal: (**a**) β’ = 16433 at Point A, (**b**) β’ = 16790 at Point B, (**c**) β’ = 17019 at Point C.

As already mentioned, when conducting nonlinear ultrasonic testing to assess the material nonlinearity, it is crucial to know what other nonlinearities are embedded in the measurements. In this work, the nonlinearity of the sensing system is investigated by analyzing the signal at points A–D in the sensing system (Figure 3a). A sequence of A-scans and frequency spectra obtained at points A–D for a single frequency toneburst having central frequency *f_o_* = 5 MHz are shown in Figure 5. The frequency spectrum in Figure 5a indicates that in addition to the primary frequency, higher harmonics are sent from the gated amplifier to the transducer. The nonlinearity of the transducer output signal is determined by the transducer response characteristics such as its nonlinearity and bandwidth. Figure 5b shows the signal received on the surface of the transducer, in which we observe the suppression of the third harmonic (relative to Figure 5a). Ultrasonic gel couples the transducer to the Plexiglas wedge. The signal amplitude is reduced due to impedance mismatch and attenuation in the wedge. Nonlinearity of the wedge and possible contact nonlinearity between the transducer and the wedge increase the higher harmonic content of the signal in Figure 5c. The relative nonlinearity parameter measured using linear regression at Points A–C is shown in Figure 6. The nonlinearity at these points is entirely from the sensing system. We observe that although the signal amplitude reduces at each stage, the nonlinearity of the signal is increased by 2.17% at Point B and by 3.57% at Point C.

The signal received at Point D is shown in Figure 5d. This signal contains all of the nonlinearities as the signal received at Point C as well as the nonlinearity associated with Rayleigh wave propagating 40 mm in the aluminum block. The nonlinearity associated with Rayleigh wave propagation is due to the material nonlinearity as well as the surface roughness. It may be possible to directly quantify the nonlinearity associated with Rayleigh wave propagation by subtracting the Point C spectrum from the Point D spectrum after they have been normalized with respect to the primary frequency. However, doing so presumes no interaction between the system nonlinearity, the material nonlinearity, and the surface roughness. We do not perform this subtraction in the remainder of this work because all measurements contain the same system nonlinearities. Therefore, we are interested in changes in the nonlinearity.

Alternate versions of Figure 5 and Figure 6 using a normalized dB scale are included in the Supplemental Materials (Figures S1 and S2 respectively). The normalized dB scale provides a nice visualization of changes in the second harmonic amplitudes due to the system nonlinearity at different points in the generation of nonlinear Rayleigh waves.

### 3.2. Nonlinear Rayleigh Wave Mixing Methods

Two different methods for dual-frequency Rayleigh wave excitation for wave mixing are investigated from the viewpoint of the system nonlinearities. The first approach uses a single transducer excited by a dual-frequency toneburst. Figure 1 shows the test setup to study the response of the transducer as received by the laser interferometer. The mutual interaction of waves at primary frequencies *f_a_* = 3.2 MHz and *f_b_* = 3.84 MHz generated by a single transducer is studied. The second-order frequencies are: $f_{b}-f_{a}=0.64$ MHz, $2f_{a}=6.4$ MHz, $f_{b}+f_{a}=7.04$ MHz, and $2f_{b}=7.68$ MHz. 

**Figure 7:**
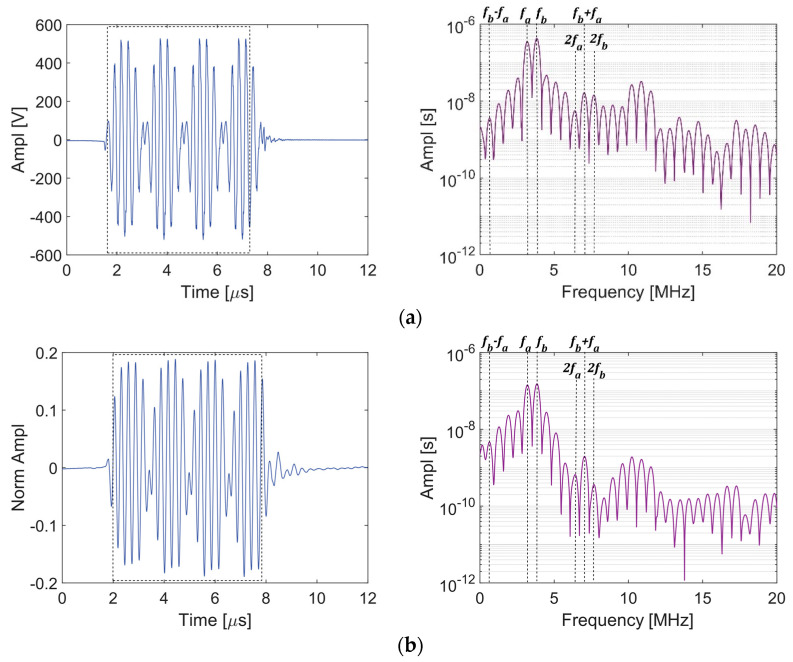
A-scan and frequency spectrum given a dual-frequency excitation (*f_a_* = 3.2 MHz and *f_b_* = 3.8 MHz) excitation at the 75% output level: (**a**) Point A, (**b**) Point B.

Figure 7 shows the A-scans and the frequency spectra for the signals received at Point A (output of amplifier) and Point B (surface of the transducer). The four packets observed in the A-scans indicate the presence of two excitation frequencies (*f_a_* and *f_b_*). The two excitation frequencies, the corresponding second harmonics, and the sum and difference frequency peaks are marked in the frequency spectra. The frequency spectrum from Point A shows that the dual-frequency signal undergoes modulation before getting to the transducer. Thus, the basic premise for mixing waves is violated—i.e., there is energy present at the sum and difference frequencies that is not associated with the nonlinearity of the waveguide material. The higher harmonics generated due to the nonlinearity in the system complicate the measurement of the material nonlinearity. Several other high amplitude peaks can also be observed in the frequency spectrum. This is a typical phenomenon observed when two frequencies are mixed in a nonlinear device (amplifier) and is known as intermodulation distortion, wherein the higher harmonics of frequencies that are integral multiples of the two excitation frequencies are generated due to the electrical system nonlinearity. These harmonics can be represented as $\left|nf_{a}+mf_{b}\right|$, where n and m are integers. The sum |n|+|m| is referred to as the order of the distortion. Thus, additional peaks at other combinational frequencies such as 2*f_a_* + *f_b_*, 2*f_a_* − *f_b_*, *f_a_* + 2*f_b_*, 3*f_a_* − 2*f_b_* are also observed in the frequency spectrum.

**Figure 8:**
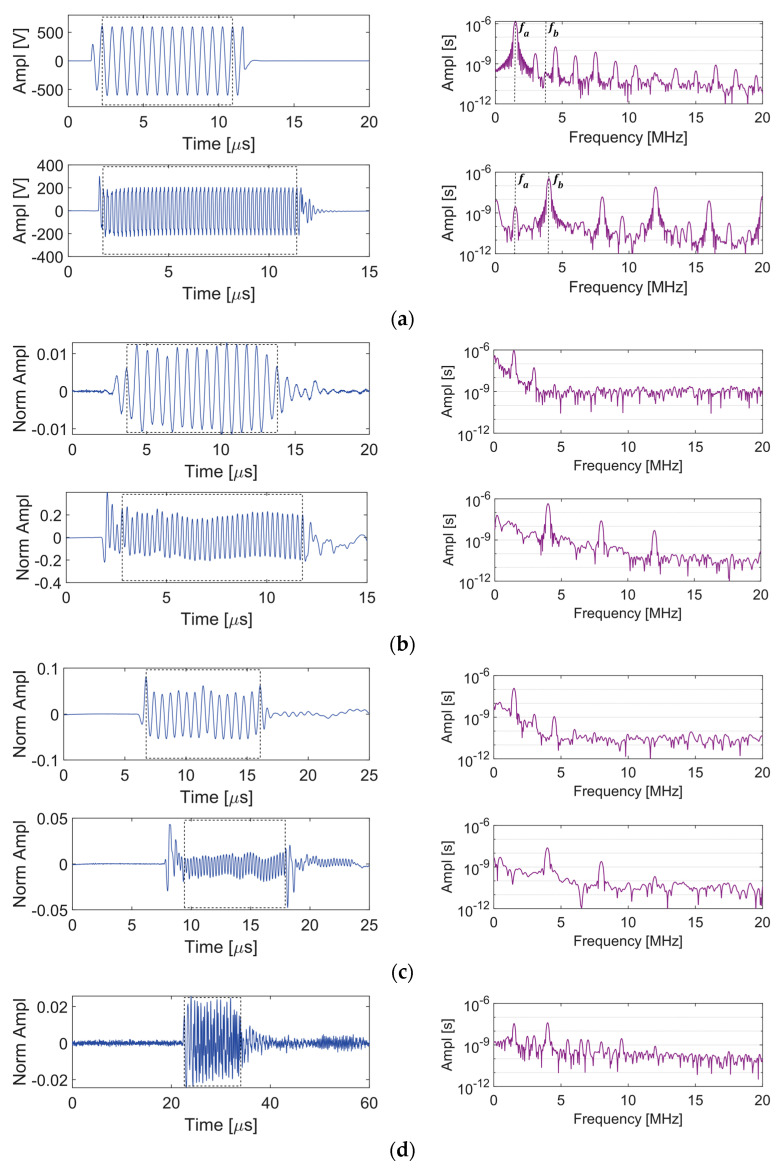
A-scan and frequency spectrum given toneburst excitations to adjacent transducers at the 75% output level: (**a**) Point A, (**b**) Point B, (**c**) Point C, (**d**) Point D.

The alternative to sending a dual-frequency signal to a single transducer is to send separate signals to two adjacent transducers. The 2.25 and 5 MHz transducers are placed on side-by-side wedges and the primary frequencies *f_a_* = 1.5 MHz and *f_b_* = 4.0 MHz are generated by the two gated amplifier channels. The second-order frequencies are: $f_{b}-f_{a}=2.5$ MHz, $2f_{a}=3.0$ MHz, $f_{b}+f_{a}=5.5$ MHz, and $2f_{b}=8.0$ MHz. The A-scans and frequency spectra for Points A-D are shown in Figure 8. Figure 8a shows that amplifier Channel 1 outputs *f_a_* and its higher harmonics only, while Channel 2 outputs *f_b_* and its higher harmonics in addition to a small peak at *f_a_*. However, the spurious peak at *f_a_* is not observed in the signal sent from the transducer in Figure 8b, perhaps due to limitations of the bandwidth of the transducer (although this was not investigated). Figure 8c presents the signals obtained on the wedges and their frequency spectra. Finally, the mixing Rayleigh waves are received at a point located 40 mm from the adjacent wedges and the signal is shown in Figure 8d. Unlike when a dual frequency signal was sent to a single transducer (Figure 7), where the frequency spectrum consists of many equal-width lobes, the frequency spectrum in Figure 8d consists of distinct peaks at the primary and second order frequencies.

### 3.3. Surface Roughness Effects on Rayleigh Wave Interactions

**Figure 9:**
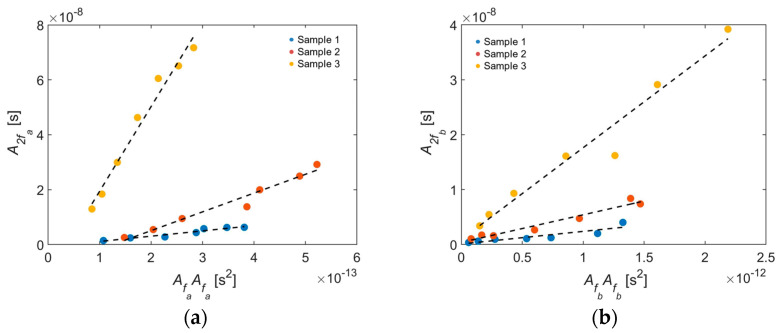
Second-order spectral amplitudes for self-interaction plotted as a function of the square of the primary frequency amplitudes for (**a**) 2*f_a_* and (**b**) 2*f_b_*. *f_a_* = 1.5 MHz and *f_b_* = 4.0 MHz.

**Figure 10:**
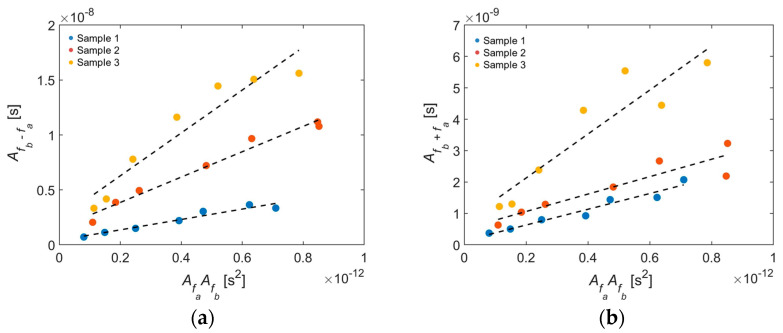
Second-order spectral amplitudes for mutual interaction plotted as a function of the product of the primary frequency amplitudes for (**a**) *f_b−a_* and (**b**) *f_b+a_*. *f_a_* = 1.5 MHz and *f_b_* = 4.0 MHz.

On each sample the adjacent wedge transducers sent Rayleigh waves that were received by the laser interferometer. From the frequency spectrum the peaks at the primary and secondary frequencies were determined. Figure 9 plots the amplitude peak at the second harmonic frequency ($A_{2f_{a}}$ or $A_{2f_{b}}$) versus the square of the amplitude peak at the corresponding primary frequency ($A_{f_{a}}A_{f_{a}}$ or $A_{f_{b}}A_{f_{b}}$, respectively). Likewise, Figure 10 plots the amplitude peak at the combinational harmonic frequency ($A_{f_{b-a}}$ and $A_{f_{b+a}}$) versus the product of the amplitude peaks at the corresponding primary frequencies ($A_{f_{a}}A_{f_{b}}$). The relative nonlinearity parameters (Equation (2)) were regressed to the results shown in Figure 9 and Figure 10 for self-interaction and mutual interaction, respectively. The relative nonlinearity parameters for each sample and secondary frequency are tabulated in Table 3. The relative nonlinearity parameter increases with surface roughness from Sample 1 to Sample 2 to Sample 3. The roughness magnification factors for Sample 2 relative to Sample 1 and for Sample 3 relative to Sample 1 were computed and are also given in Table 3. Magnification factors range from 1.10 to 2.44 for the moderate sample and from 2.79 to 16.0 for the rough sample, both taken relative to the smooth sample. The magnification factor is larger for self-interaction than mutual interaction, with the exception of Sample 2 at *f_2a_*, which could be due to the larger system nonlinearity for the second harmonic relative to the sum and difference frequencies. The magnification factor is the smallest for *f_b + a_*. Note that the largest average roughness value (3.992 μm) is two orders of magnitude smaller than the smallest wavelength (360 μm). The increase in relative nonlinearity parameter due to surface roughness is consistent with the results of Liu et al. [12].

In the Introduction we noted that surface roughness causes scattering, which in turn causes attenuation. Other researchers have corrected the nonlinearity parameter for attenuation [38], which leads us to assess whether the variations in the relative nonlinearity parameter in Table 3 are due to the attenuation caused by surface roughness. Let us reconsider Equation (2) for the relative nonlinearity parameter for a material having attenuation that increases with frequency. In comparison with a lossless material, a lossy material will have a lower $\beta^{\prime }$ for the sum frequency, but a higher $\beta^{\prime }$ for the difference frequency (if the difference is less than *f_a_*). Likewise, a lossy material will have a lower $\beta^{\prime }$ for second harmonics. Therefore, by increasing the attenuation and with all other material parameters remaining unchanged, $\beta^{\prime }$ should decrease. By this argument, the increasing $\beta^{\prime }$ with surface roughness observed in Table 3 is not associated with attenuation. We will go through the analysis to verify that the argument is indeed correct. Therefore, the attenuation of Rayleigh waves at different frequencies is characterized in the next section.

### 3.4. Effect of Attenuation

**Figure 11:**
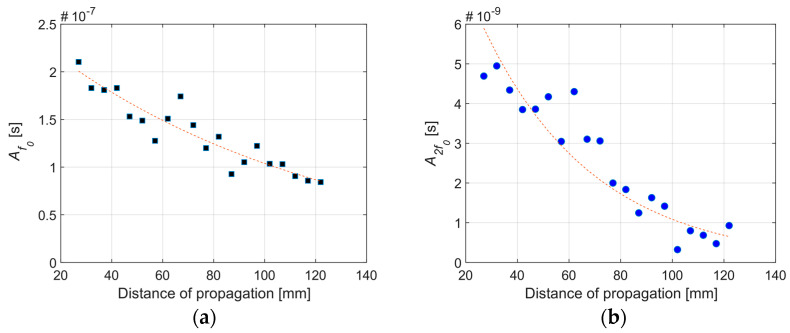
Sample attenuation curves for (**a**) primary (2 MHz) and (**b**) secondary (4 MHz) waves from Sample 3.

**Figure 12:**
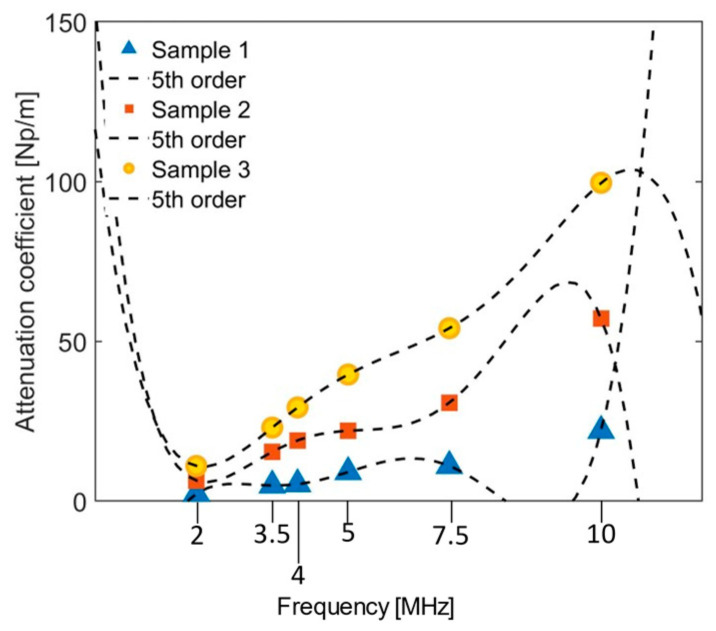
Frequency dependence of the attenuation coefficients for Rayleigh waves on aluminum block.

Let the attenuation of the Rayleigh waves be given by

(3) $$A_{n}={\left(A_{n}\right)}_{0}e^{-\alpha_{n}x}$$

where $A_{n}$ is the wave amplitude including attenuation, ${\left(A_{n}\right)}_{0}$ is the initial amplitude of the wave, $\alpha_{n}$ is the attenuation coefficient, x is the propagation distance, n=1 for the primary frequency and n=2 for the second harmonic frequency. Attenuation coefficients are determined for the primary frequencies (2 MHz, 3.5 MHz, and 5 MHz) and the corresponding second harmonic frequencies (4 MHz, 7 MHz, and 10 MHz, respectively) by conducting a linear scan along the propagation path of the Rayleigh waves for the three samples. At each position in the scan the FFT is computed from the received A-scan in order to determine the amplitudes $A_{1}$ and $A_{2}$ corresponding to the primary frequency and the second harmonic frequency, respectively. Figure 11 shows example attenuation curves obtained for the Rayleigh waves with primary frequency *f*_0_ = 2 MHz and second harmonic frequency 2*f*_0_ propagating on Sample 3 (the full set of attenuation curves are provided in the Supplementary Materials). Figure 12 shows the frequency-dependence of the attenuation coefficients for the three blocks is well-represented as 5th order. The regressed attenuation coefficients are seen to increase with increasing frequency and surface roughness in Table 4.

On the other hand, the amplitude of the second harmonic is cumulative and increases linearly with propagation distance [34,39]

(4) $$A_{2}=\frac{1}{8}\beta A_{1}^{2}k^{2}x$$

Using Equations (3) and (4), the spatial change in the second harmonic wave amplitude due to distortion and attenuation can be expressed as

(5) $$\frac{dA_{2}}{dx}=\frac{1}{8}\beta A_{1}^{2}k^{2}-\alpha_{2}A_{2}$$

which is a first order ordinary differential equation that can be solved by imposing the initial condition that $A_{2}=0$ at x=0. Substituting Equation (3) in for $A_{1}$, the solution (due to Hikata and Elbaum [40], see also Cantrell [34]) can be written as

(6) $$A_{2}=\frac{1}{8}\beta k^{2}{\left(A_{1}\right)}_{0}^{2}\left[\frac{\exp\left(-2\alpha_{1}x\right)-\exp\left(-2\alpha_{2}x\right)}{\alpha_{2}-2\alpha_{1}}\right]$$

Let $\beta \prime_{meas}$ be given by Equation (1) and use that to solve for the attenuation-corrected relative nonlinearity parameter

(7) $$\beta \prime_{corrected}=\beta \prime_{meas}\frac{x\left(\alpha_{2}-2\alpha_{1}\right)}{1-\exp\left[-x\left(\alpha_{2}-2\alpha_{1}\right)\right]}$$

**Figure 13:**
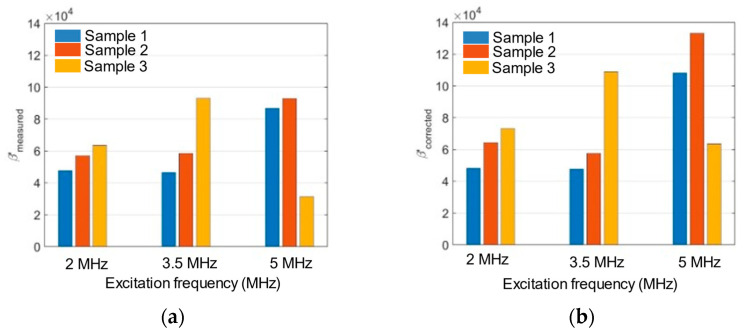
Relative nonlinearity parameter for each excitation frequency: (**a**) measured, (**b**) corrected.

The relative nonlinearity parameters are obtained using the experimental method described in Section 2.4 for each sample and frequency. Figure 13 shows bar charts of the relative nonlinearity parameter for each excitation frequency. In Figure 13a, $\beta \prime_{meas}$ is directly from the measurements, while in Figure 13b $\beta \prime_{corrected}$ is corrected for attenuation by using Equation (7).

In Figure 13a, we observe that for the 2 MHz and 3.5 MHz excitation frequencies, the $\beta \prime_{meas}$ increases with the increase in the surface roughness. This observation is consistent with the effect observed for the mutual interaction study described in the previous section. For the 5 MHz excitation frequency, the relative nonlinearity parameter increases from Sample 1 to 2 but decreases for Sample 3. We attribute the reduction in the relative nonlinearity parameter for Sample 3 to the dominance of the attenuation effects over the harmonic generation, since the attenuation effects are more pronounced at higher frequency and surface roughness. $\beta \prime_{meas}$ generally increases with frequency until attenuation overwhelms the nonlinearity.

Table 5 provides the correction factors (fraction on right-hand side of Equation (7)) computed for each excitation frequency and surface roughness. The correction factors range from 0.9841 to 2.0347. We note that the correction factors are generally higher for both higher frequency and larger surface roughness, except for a slight decrease observed for 3.5 MHz excitation on Sample 2. If the attenuation correction worked as intended, the $\beta \prime_{corrected}$ for a prescribed frequency would have been the same for all three samples. Clearly, it is not. Moreover, attenuation should make $\beta \prime_{corrected}<\beta \prime_{meas}$, and the correction factor less than one. Clearly, it is not. These results suggest that the surface roughness effects on the relative nonlinearity parameter cannot be corrected by attenuation. In general, we infer that the surface roughness influences the relative nonlinearity parameter and its effect depends on the average asperity height and the wavelength of the Rayleigh waves.

## 4. Discussion

Our experimental results in Table 3 and Figure 13 show that the variation of average asperity height (*P_a_*) from 0.027–3.992 μm along an aluminum surface has a substantial effect on the distortion of Rayleigh waves for excitation frequencies between 1.5 and 5 MHz. These asperities are small compared to the wavelengths. The largest Rayleigh wavelength is 1.9 mm at 1.5 MHz, while the smallest wavelength is 0.29 mm for the second harmonic at 10 MHz. Here, we quantify wave distortion through the relative nonlinearity parameter given in Equation (2). While surface roughness increases the attenuation of Rayleigh waves relative to a smooth surface, increased attenuation actually decreases the wave distortion. In contrast, Table 5 indicates that the roughness-induced attenuation actually increases the nonlinearity parameter.

Rayleigh wave distortion (nonlinearity) is useful for nondestructively assessing structural integrity and material degradation. However, these results strongly suggest that in order to use Rayleigh waves to assess material nonlinearity, we need to have a good understanding of the nonlinearities associated with surface roughness in addition to those associated with the sensing system. The interaction between the material nonlinearity and the surface roughness is entirely different from its interaction with the sensing system because material and surface nonlinearities occur in parallel, while material and sensing system nonlinearities occur in series.

These experiments were conducted due to our interest in using Rayleigh waves to monitor the additive manufacturing process. However, the roughness of metal surfaces during powder bed fusion and directed energy deposition processes is significantly larger than it was here. Current research is investigating this challenging problem. A future research direction is to explore the physics underlying the Rayleigh wave distortion associated with small surface asperities.

## 5. Conclusions

Nonlinear Rayleigh wave measurements aimed at correlating with nonlinear material response are complicated by sensing system nonlinearities and surface roughness. The sensing system nonlinearities are quantified by obtaining signals at four generation stages: the output of the amplifier, the surface of the transducer, on the acrylic wedge, and the surface of the specimen. Wave mixing experiments enable material nonlinearities to be received at frequencies free from sensing system nonlinearities only if separate transducers are used to generate the waves that mix only in the waveguide.

The effects of surface roughness on the nonlinearity (distortion) of Rayleigh waves that are self-interacting or mutually interacting were investigated. The experimentally determined relative nonlinearity parameter exhibits a frequency-dependent relationship with the surface roughness. The variation in the relative nonlinearity parameter for different surface roughness is not correctable through attenuation and needs to be investigated further to understand the physics associated with roughness increasing the wave distortion.

## Figures and Tables

**Table 1:** 3D and 1D surface profiles for the three aluminum test blocks.

Sample	3D Surface Profile	1D Surface Profile
1 Smooth	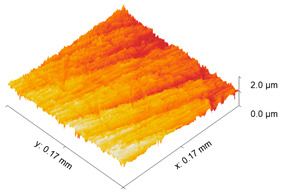	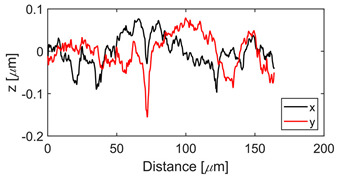
2 Moderate	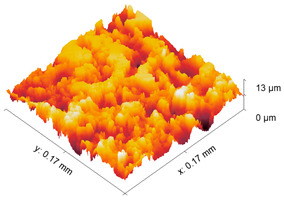	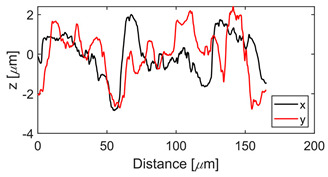
3 Rough	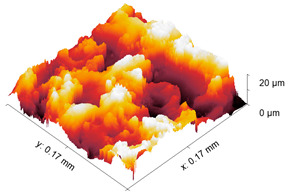	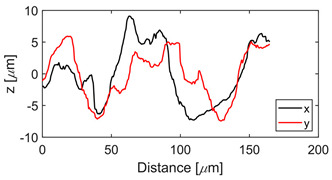

**Table 2:** Surface roughness parameters for the three aluminum test blocks.

Sample	Linear Roughness Parameters (ISO 4287): x-Direction					
	$P_{a}$, μm		$P_{q}$, μm		$P_{t}$, μm	
1 (Smooth)	0.027		0.034		0.173	
2 (Moderate)	0.872		1.081		4.849	
3 (Rough)	3.992		4.649		16.403	
**Sample**	**Linear Roughness Parameters (ISO 4287): y-Direction**					
	$P_{a}$ , μ **m**		$P_{q}$ , μ **m**		$P_{t}$ , μ **m**	
1 (Smooth)	0.033		0.040		0.234	
2 (Moderate)	1.034		1.304		5.178	
3 (Rough)	3.410		3.923		13.365	
**Sample**	**Areal Roughness Parameters (ISO 25178-2)**					
	$S_{a}$ , μ **m**	$S_{q}$ , μ **m**		$S_{z}$ , μ **m**		***S_dq_***
1 (Smooth)	0.0831	0.105		0.865		0.220
2 (Moderate)	1.642	1.993		12.94		1.852
3 (Rough)	4.349	5.118		20.450		2.832

**Table 3:** Relative nonlinearity parameter obtained from wave mixing test with *f_a_* = 1.5 MHz and *f_b_* = 4.0 MHz.

Secondary Frequency (MHz)	Relative Nonlinearity Parameter			Roughness Magnification Factor	
	Sample 1 Smooth	Sample 2 Moderate	Sample 3 Rough	Sample 2/1	Sample 3/1
*f_b−a_* = 2.5	4725	11,545	19,514	2.44	4.13
*f_2a_* = 3.0	19,301	33,675	308,435	1.74	16.0
*f_b+a_* = 5.5	2509	2774	7003	1.10	2.79
*f_2b_* = 8.0	2298	5015	16,717	2.18	7.27

**Table 4:** Attenuation coefficient α in Np/m for primary and second harmonic Rayleigh waves.

Sample	Roughness		*f*_o_ = 2.0 MHz	*f*_o_ = 3.5 MHz	*f*_o_ = 5.0 MHz
1	Smooth	*f*_o_ 2*f*_o_	2.3 5.3	4.9 11.0	5.3 22.0
2	Moderate	*f*_o_ 2*f*_o_	6.4 19.0	15.4 30.8	19.0 57.2
3	Rough	*f*_o_ 2*f*_o_	11.0 29.3	23.0 54.1	29.3 99.6

**Table 5:** Relative nonlinearity parameter correction factor (Equation (7)).

Sample	Roughness	*f*_o_ = 2.0 MHz	*f*_o_ = 3.5 MHz	*f*_o_ = 5.0 MHz
1	Smooth	1.0141	1.0242	1.2453
2	Moderate	1.1290	0.9841	1.4327
3	Rough	1.1531	1.1707	2.0347

## Data Availability

The data in this study are available in the article and Supplementary Materials.

## Supplementary Materials

The following are available online at https://www.mdpi.com/article/10.3390/s21165495/s1, Figure S1. A-scan and frequency spectrum given a toneburst excitation (*f_o_* = 5 MHz) at the 75% output level: (a) Point A, (b) Point B, (c) Point C, and (d) Point D, Figure S2. Linear regression to determine the relative nonlinearity parameter for the measurement system given a 5 MHz signal: (a) β’ = 0.3942 at Point A, (b) β’ = 0.5370 at Point B, (c) β’ = 0.7478 at Point C., Figure S3. Attenuation curves for Rayleigh waves propagating on Samples 1–3 having primary frequency (a) 2 MHz, (b) 3.5 MHz, (c) 5 MHz.
