# Supplementary material for: Surface Roughness Effects on Self-Interacting and Mutually Interacting Rayleigh Waves
Source: Sensors (Basel). 2021 Aug 15;21(16):5495. doi: 10.3390/s21165495 (PMC8400126; doi:10.3390/s21165495)
Supplement: Supplementary file 1 [file sensors-21-05495-s001.zip › sensors-1301150-supplementary.pdf]

Supplemental Materials

Figures S1 and S2 are alternate versions of Figures 4 and 5 with the frequency spectrum plotted on the dB scale. In Figure S1, the frequency domain amplitudes are normalized with respect to the peak spectral amplitude at the primary frequency. The dB scale allows better visualization of the changes in the second harmonic amplitudes due to system nonlinearity at Points A-D. In Figure S2, we want to point out that the values for the relative nonlinearity parameters obtained at Points A-C in dB scale are different from those obtained in the linear scale due to the normalization. Figure S3 shows the data to which the attenuation coefficients are fit.

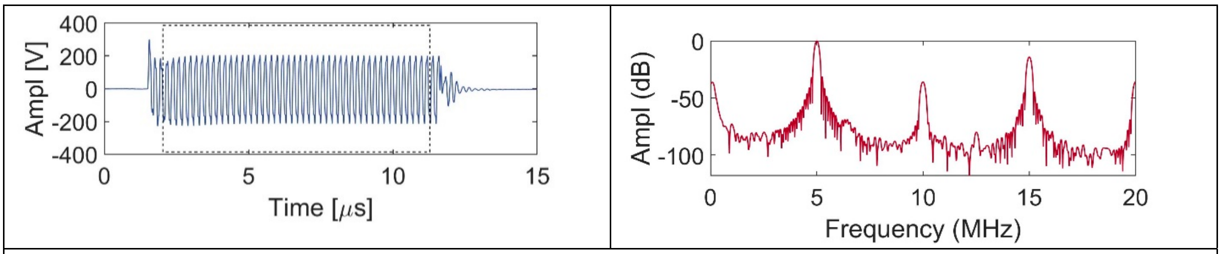

(**a**) Point A: received from amplifier

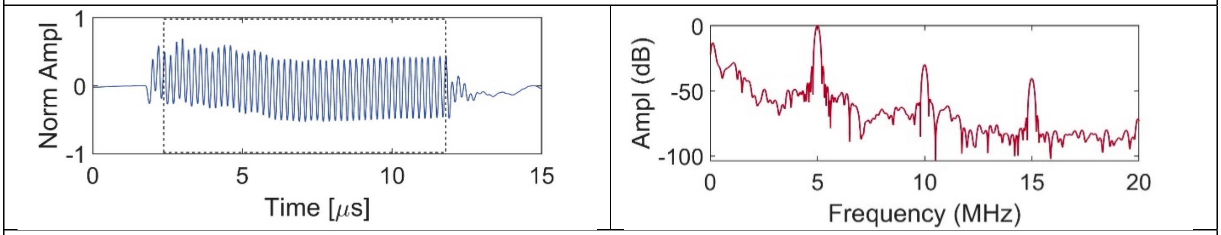

(**b**) Point B: received from transducer surface

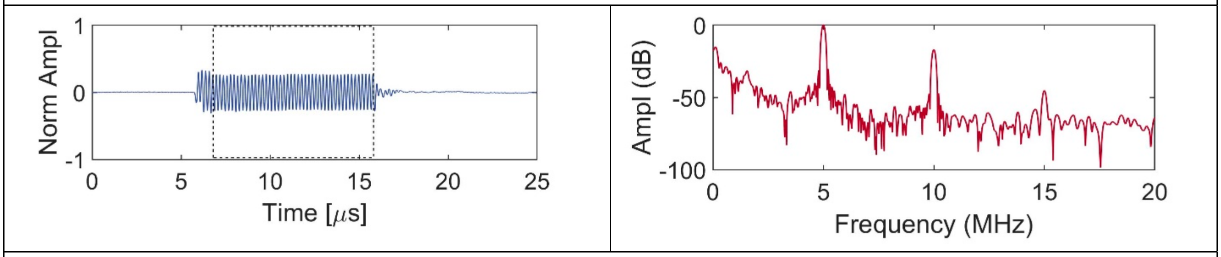

(**c**) Point C: received from wedge

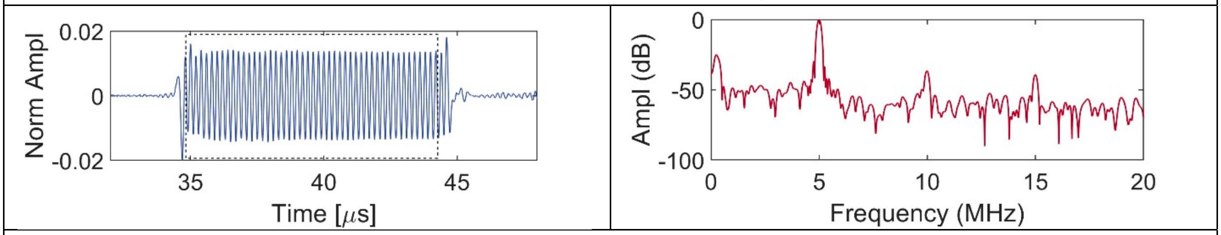

(**d**) Point D: received from Sample 1

Figure S1. A-scan and frequency spectrum given a toneburst excitation (f_o = 5 MHz) at the 75% output level: (a) Point A, (b) Point B, (c) Point C, and (d) Point D.

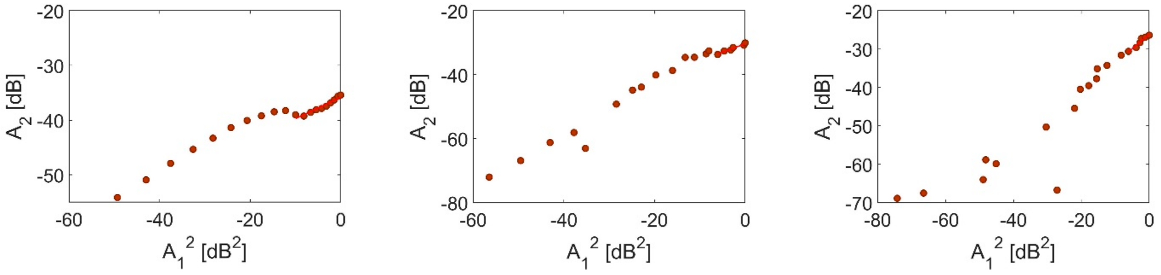

Figure S2. Linear regression to determine the relative nonlinearity parameter for the measurement system given a 5 MHz signal: (a) b′ = 0.3942 at Point A , (b) b ′ = 0.5370 at Point B, (c) b ′ = 0.7478 at Point C.

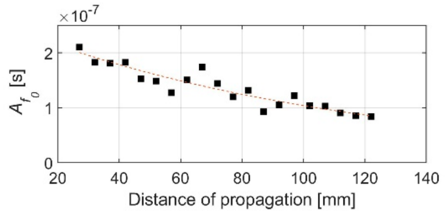

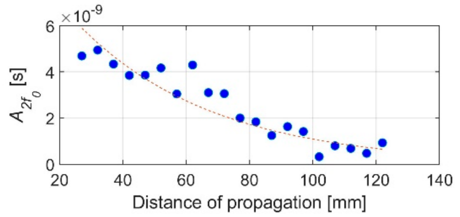

(i) Sample 1

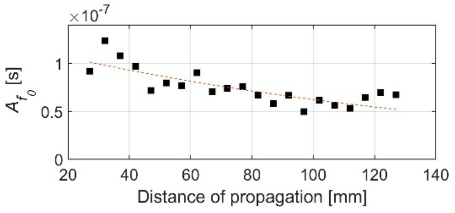

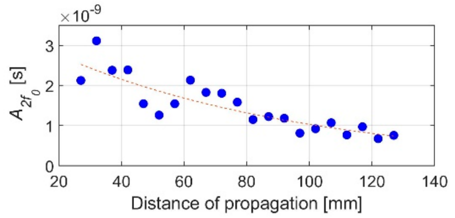

(ii) Sample 2

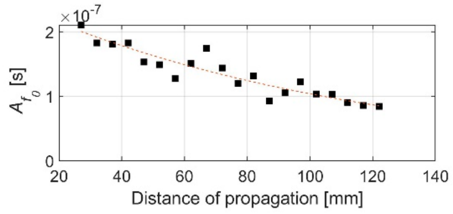

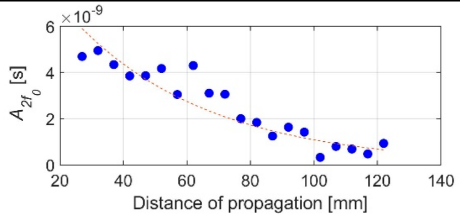

(iii) Sample 3

(**a**) Attenuation curves for 2 MHz excitation frequency

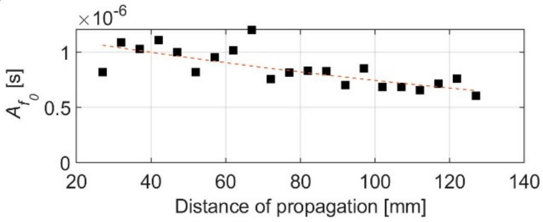

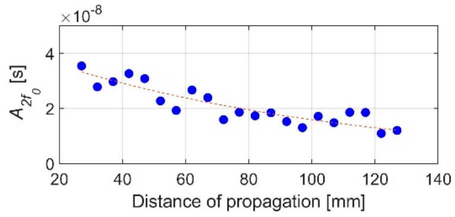

(i) Sample 1

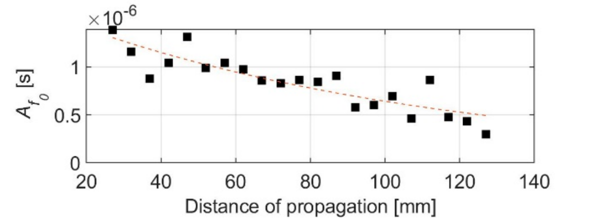

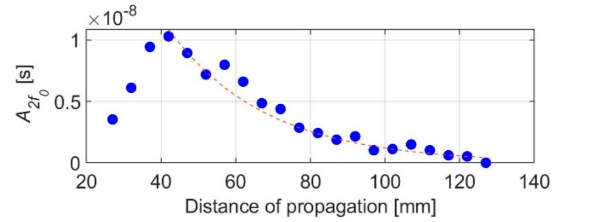

(ii) Sample 2

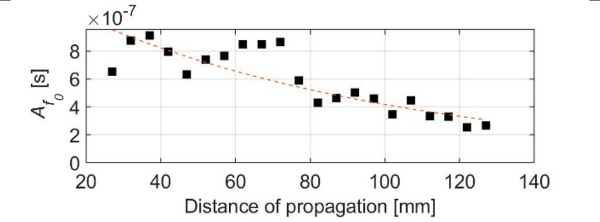

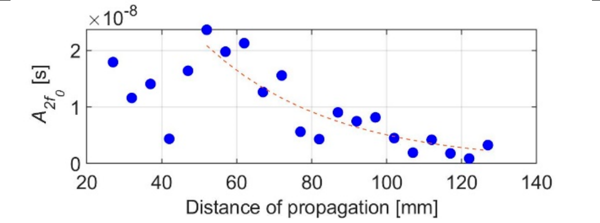

(iii) Sample 3

(**b**) Attenuation curves for 3.5 MHz excitation frequency

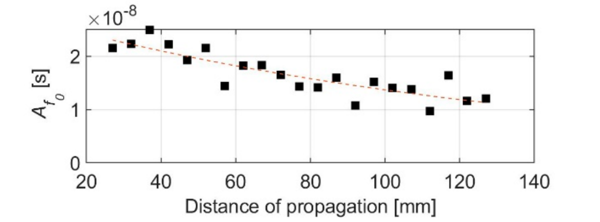

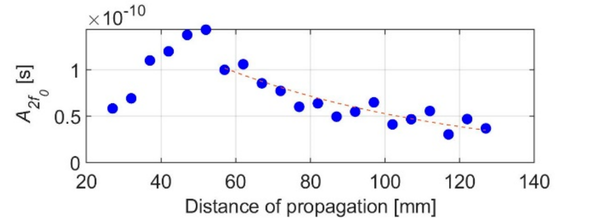

(i) Sample 1

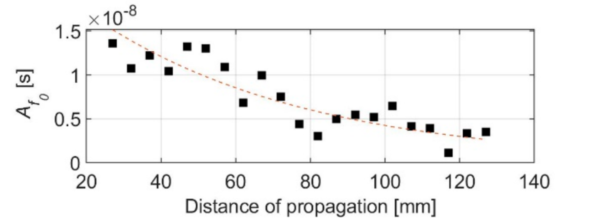

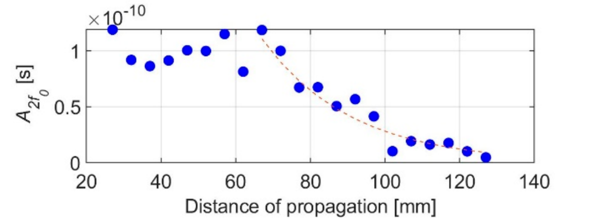

(ii) Sample 2

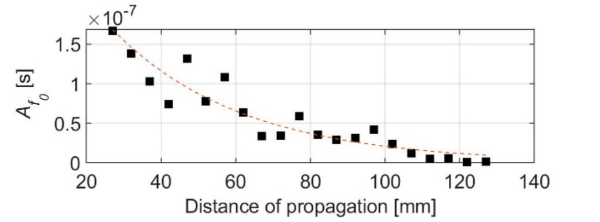

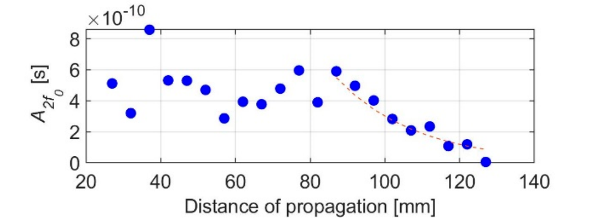

(iii) Sample 3

(**c**) Attenuation curves for 5 MHz excitation frequency

Figure S3. Attenuation curves for Rayleigh waves propagating on Samples 1-3 having primary frequency (**a**) 2 MHz, (**b**) 3.5 MHz, and (**c**) 5 MHz.
